# Mosquito-Specific Viruses—Transmission and Interaction

**DOI:** 10.3390/v11090873

**Published:** 2019-09-17

**Authors:** Eric Agboli, Mayke Leggewie, Mine Altinli, Esther Schnettler

**Affiliations:** 1 Molecular Entomology, Molecular Biology and Immunology Department, Bernhard Nocht Institute for Tropical Medicine, 20359 Hamburg, Germany; 2 Department of Epidemiology and Biostatistics, School of Public Health, University of Health and Allied Sciences, Ho PMB 31, Ghana; 3 German Centre for Infection research (DZIF), partner site Hamburg-Lübeck-Borstel-Riems, 20359 Hamburg, Germany

**Keywords:** MSV, arbovirus, horizontal transmission, vertical transmission, RNAi, vector control, vaccines

## Abstract

Mosquito-specific viruses (MSVs) are a subset of insect-specific viruses that are found to infect mosquitoes or mosquito derived cells. There has been an increase in discoveries of novel MSVs in recent years. This has expanded our understanding of viral diversity and evolution but has also sparked questions concerning the transmission of these viruses and interactions with their hosts and its microbiome. In fact, there is already evidence that MSVs interact with the immune system of their host. This is especially interesting, since mosquitoes can be infected with both MSVs and arthropod-borne (arbo) viruses of public health concern. In this review, we give an update on the different MSVs discovered so far and describe current data on their transmission and interaction with the mosquito immune system as well as the effect MSVs could have on an arboviruses-co-infection. Lastly, we discuss potential uses of these viruses, including vector and transmission control.

## 1. Discovery of Different Mosquito-Specific Viruses (MSVs)

Insect-specific viruses (ISVs) are emerging viruses that are restricted to insects and are unable to replicate in mammalian cells [1]. A sub-set of ISVs are the mosquito-specific viruses (MSVs), that have so far only been discovered and successfully cultured in mosquitoes or mosquito cell lines. Notably, there are viruses that are presumed to be MSVs although there is still a lack of experimental data involving their ability to infect mammals. In recent years, there has been an increase in discoveries of MSVs [2,3]. Interestingly, many of these viruses taxonomically cluster very close to arthropod-borne (arbo) viruses, of which many are of public health concern [2].

In this review we discuss MSVs, their discovery, transmission, possible interference during co-infections with other viruses like arboviruses and their potential uses. 

### 1.1. The Discovery

Many virus families, such as *Parvoviridae*, *Baculoviridae* and *Iridoviridae*, causing apparent symptoms or mortality in mosquito larvae, have been discovered and studied as early as the 1960s. However, the discovery of MSVs (especially the ones not causing obvious symptoms in mosquitoes), significantly increased with the application of next generation sequencing (NGS) techniques in virus discovery [4,5]. Continually isolated and identified in the last decade, MSVs are now found in many diverse virus families (Table 1), based on their characteristics and phylogenetic studies. Here we describe some of these families, where most MSVs have been found, as well as the ones where most arboviruses belong. 

#### 1.1.1. Flaviviridae

These viruses are enveloped and possess a single-stranded, positive-sense RNA genome of approximately 11kb [6,7]. The first MSV identified, isolated and characterized is the Cell-fusing Agent Virus (CFAV). Characterization of this virus was done in *Aedes aegypti* cell lines providing a conspicuous cytopathic effect (CPE) but was unable to infect vertebrate cells [8]. Most MSVs discovered today have been grouped into the *Flaviviridae* family. Insect-specific flaviviruses (ISFVs) are placed in two groups (Classical ISFV and Dual-host ISFV) based on phylogenetics [9]. Classical ISFVs, such as CFAV and Kamiti River virus (KRV), are phylogenetically distinct from all previously known flaviviruses [9]. Dual-host ISFVs are phylogenetically linked to the arthropod borne and unknown vector flaviviruses such as Chaoyang virus (CHAOV) and Donggang virus (DONV) [9]. 

#### 1.1.2. Togaviridae

Alphaviruses are icosahedral, small, enveloped viruses with single-stranded, positive-sense RNA genomes of 10–12 kb [10]. Members of this virus family include the Eilat virus (EILV) and Tai Forest Alphavirus (TFAV). EILV was the first MSV Alphavirus isolated from a pool of *Anopheles coustani* mosquitoes from the Negev desert of Israel. Phylogenetic analyses placed EILV as a sister to the Western equine encephalitis antigenic complex within the main clade of mosquito-borne alphaviruses. [11]. Amongst the MSV, *Togaviridae* has the smallest number of viruses discovered, in contrast to other virus families.

#### 1.1.3. Bunyaviridae

Viruses in this family are enveloped and have a linear, segmented, single-stranded RNA genome. The viral genome comprises three unique molecules of negative or ambi-sense ssRNA, designated L (large), M (medium) and S (small), which total 11–19 kb [12]. Most of the MSVs in this family were identified in Africa and mostly associated with *Culex* mosquitoes [13,14,15]. Badu virus (BADUV) was the first Bunyavirus identified based on time of collection of mosquito specimen from the field [16]. BADUV was shown to replicate in mosquito cells originating from both *Culex* and *Aedes*. [16]. 

#### 1.1.4. Mesoniviridae

This recently established family in the order *Nidovirales* possesses a linear single-stranded positive-sense RNA genome (13–20 kb) and an enveloped capsid [17]. The family was established in 2012 to accommodate Cavally virus (CAVV) discovered in *Culex* mosquitoes captured in Côte d’Ivoire, 2004 [18]. Yichang virus (YCV) is the latest discovery in this family. Wang and colleagues isolated this virus in *Culex* mosquitoes in Hubei, China. YCV appears as a spherical particle with a diameter of ∼80 nm and large club-shaped projections. [19]. Viruses in this family have a broad host range and an extensive geographical distribution.

## 2. Interaction of Mosquito-Specific Viruses with Arboviruses

As discussed above, MSVs have a strict host tropism and are incapable to replicate in mammalian cells. Mosquitoes can however be infected with both MSVs and arboviruses simultaneously and it has been shown already that MSVs may affect the mosquitoes’ ability to acquire, maintain and transmit these viruses (i.e. vector competence) [1,75]. This section of the review describes the interaction of MSVs with arboviruses in cell culture and live mosquitoes (summarized in Table 2).

### 2.1. Flaviviruses

It was shown in in vivo experiments with *Culex pipiens* mosquitoes persistently infected with Culex Flavivirus (CxFV) that this MSV is able to suppress the replication of West Nile Virus (WNV) [76]. The results also suggest that the presence of CxFV may impact the intensity of enzootic transmission of WNV and the risk of human exposure [76]. In another experiment, CxFV had no effect on the replication of WNV using injected *Culex quinquefasciatus* mosquitoes [77]. Furthermore, the CxFV-infection in *Culex pipiens* did not affect the transmission of Rift Valley Fever virus (RVFV). This implies that CxFV existing in field-collected *Culex pipiens* populations does not affect their vector competence for RVFV [78]. Overall, the effect of CxFV on investigated arboviruses replication depends on the infection route and mosquito species.

Palm Creek virus (PCV) suppressed replication of WNV and Murray Valley Encephalitis Virus (MVEV) by 10–43 fold at 48 hours post-infection in *Aedes albopictus* derived mosquito cells (C6/36). Interestingly, no inhibitory effect of PCV infection was observed for the alphavirus Ross River virus, suggesting the possibility of superinfection exclusion between MSVs and arthropod-borne viruses belonging to the same family [21]. Other investigations with PCV also showed that persistent infection of PCV has no inhibitory effect on the replication of WNV in vivo [79]. 

Nhumirim virus (NHUV) suppressed the replication of WNV and also blocks the transmission in *Culex quinquefasciatus* mosquitoes [80,81]. The first investigation was performed by Kenney and colleagues using three arboviruses: WNV, St Louis Encephalitis virus (SLEV) and Japanese Encephalitis Virus (JEV) [80]. These in vitro co-infection experiments showed that prior or concurrent infection of *Aedes albopictus* derived mosquito cells (C6/36) with NHUV resulted in a significant reduction in virus production of WNV, SLEV and JEV. The inhibitory effect was most effective against WNV (>10^6^ fold peak titre) and SLEV (>10^4^ fold peak titre) [80]. The latest NHUV study involves CHIKV, Zika Virus (ZIKV) and Dengue Virus (DENV) [82]. In the study, NHUV suppressed the replication of ZIKV and DENV-2 but not CHIKV in *Aedes albopictus* derived cells. Significant reductions in ZIKV (10^5^ fold) and DENV-2 (10^4^ fold) were observed in cells concurrently inoculated with NHUV or pre-inoculated with NHUV [82]. The authors suggest that NHUV can interfere with both midgut infection and salivary gland infection of ZIKV in *Aedes aegypti* [82]. 

Lastly, the coinfection of CFAV (Flavivirus) and Phasi charoen-like virus (PCLV; Bunyavirus) limits replication of arboviruses in *Aedes* mosquito cells [83]. Schultz and colleagues found the growth of ZIKV to be consistent in *Aedes albopictus* cells but variable in *Aedes aegypti* cell lines. They linked this finding to the observation that PCLV was present in the ZIKV-growth-variable *Aedes aegypti* cell lines but absent in *Aedes albopictus* lines. Furthermore, PCLV infection of CFAV-positive *Aedes albopictus* cells inhibited the growth of ZIKV, DENV and La Crosse virus (LACV) [83]. Data from the first CFAV study and the recent result suggest that persistently infected cell lines with mosquito-specific virus can impact arbovirus growth.

### 2.2. Alphaviruses

Active infection of *Aedes albopictus* derived cells (C7/10) with Eilat virus (EILV) reduced the titres of co-infecting viruses (SINV, VEEV, EEEV, WEEV and CHIKV) by approximately 10–10,000 fold and delayed the replication kinetics by 12–48 hours [84]. Additionally, prior in vivo EILV infection of *Aedes aegypti* mosquitoes delayed dissemination of CHIKV for 3 days [84].

## 3. Transmission Mechanisms of MSVs

Viruses can be transmitted to the host vertically, from parents to the offspring or horizontally, from the environment or via a vector. Alternatively, viruses can adopt a mixed-mode transmission involving both horizontal and vertical transmission, which seems to be the most common form of transmission of microbiota, including viruses, in nature [91]. Transmission routes can alter the outcome of the infection and play a defining role in the ecology of the virus, their spread and their maintenance in nature [92]. 

**Figure 1:**
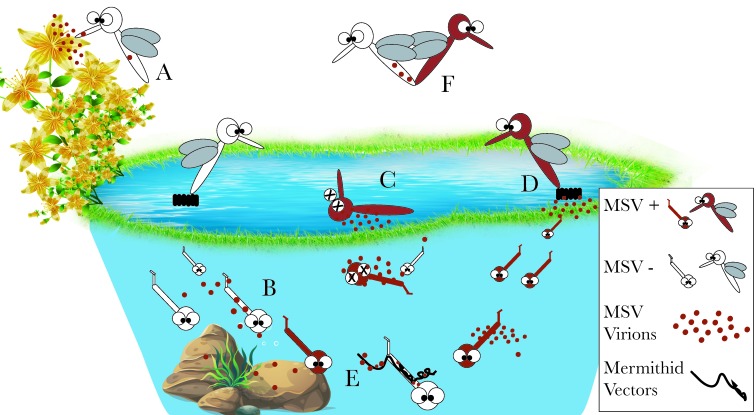
Potential transmission routes for MSVs: It is hypothesized that MSVs can be transmitted horizontally from environment to the adults through feeding on plant nectars (**A**) or to the larvae from plant material in the water or directly through the water (**B**). Infected larvae and adults might become natural reservoir for some MSVs when they die (e.g. food source) (**C**). The larvae that survive the infection can emerge as adults and potentially vertically transmit MSVs to their offspring either directly through the egg (transovarially) or indirectly by contaminating the egg surface (transovum) or the water (**D**). Some MSVs have been shown to be horizontally transmitted by parasitic mermithids, where the latter carries the MSV when they enter the larvae (**E**). MSVs can also be venereally transmitted between infected and uninfected adults in a low rate (**F**).

Arboviruses are mainly maintained in nature by horizontal transmission via an arthropod vector to a vertebrate host and they can infect and replicate in both hosts. Vertical transmission has also been reported for some arboviruses but it is commonly believed that this occurs rarely and cannot maintain the viruses alone in nature. In contrast, our knowledge of the maintenance of MSVs in nature is more limited. Nevertheless, there have been an array of experimental and field studies investigating this by looking into vertical as well as other modes of transmission (summarized in Figure 1).

### 3.1. Vertical Transmission

Mosquito specific RNA viruses are often considered vertically transmitted (Figure 1D) due to (i) their proved inability to replicate in mammalian cells, hence lacking a known vertebrate host and (ii) their presence in larvae and male adult forms, which do not feed on blood [1]. Indeed, several MSVs- including CxFV, Aedes flavivirus (AeFV), Okushiri virus and KRV- have been found in field-collected larvae, eggs or adult males [20,26,38,40,64,93,94,95,96,97,98,99]. However, experimental evidence for their vertical transmission is rare and so far limited to the *Flaviviridae* family [76]. For instance, PCV was experimentally tested for vertical transmission from females to their progeny [79]. This proved to be unsuccessful. Notably, the adults were not naturally infected but inoculated with the virus intrathoracically [79]. On the other hand, CxFV was detected in the eggs and larvae of *Cx. pipiens* lab colonies naturally infected with the virus [93,100,101]. Interestingly, investigations into the tissue tropism of the virus in the adult offspring also revealed that all tested organs (salivary glands, ovaries, testes, head, fat bodies an midguts) were positive for viral RNA [102]. Furthermore, low level venereal transmission also occurred between CxFV infected and uninfected *Cx. pipiens* males and females bidirectionally (Figure 1E) [76]. However, similar to PCV, when naïve *Cx. pipiens* were infected by injection CxFV was not vertically transmitted [102].

Moving away from RNA viruses, vertical transmission of mosquito DNA viruses is also a growing field of research. These viruses can cause high larval mortality, such as iridoviruses, baculoviruses and densoviruses, but can also be transmitted vertically in the case of covert infections with lower mortality. For instance, a low rate vertical transmission of a mosquito baculovirus Culex nigripalpus Nucleopolyhedrovirus (CuniNPV) was observed and thought to result from contamination of the larval environment or the egg rafts from the female gut [103]. Similarly, Dipteran brevidensovirus 2 (Aedes albopictus densovirus, AalDV2) can be transmitted to the offspring and the efficiency of transmission depends on the virus titre in *Aedes aegypti* females [104]. While this vertical transmission did not persist beyond the second generation in laboratory colonies, another mosquito densovirus, Dipteran Ambidensovirus 1 (Cx. pipiens densovirus) seems to persist over 20 years in *Cx. pipiens* (sl.) laboratory colonies, and vertically and transovarially transmitted at a low rate [105]. Furthermore, the amount of virus and the rate of vertical transmission was reduced by antibiotics suggesting an effect of the microbiota of the mosquitoes [105].

### 3.2. Horizontal Transmission

In addition to the vertical transmission, horizontal transmission (e.g. from water to larvae and through feeding to larvae or adult mosquitoes) can also explain the observed presence of MSVs in male adults and larvae in nature (Figure 1A–C,E). So far, mostly DNA viruses and dsRNA viruses of mosquitoes have been studied for horizontal transmission during larval stage due to the easily observable disease symptoms and resulting mortality. For example, Baculoviruses CuniNPV and UrsaNPV were able to infect *Culex* and *Uratoaenia* larvae, respectively, when Mg+ was added to the rearing water. In contrast, Ca+ inhibited the infection [103,106,107]. Similar effects of Mg+ and Ca+ on virus transmission were also observed when *Cx. quinquefasciatus* and *Ae. aegypti* larvae were experimentally infected with dsRNA viruses, *Cx. restuans* (CrCPV) and *Uranotaenia sapphirina* (UsCPV) Cytoplasmic Polyhedrosis viruses (*Reoviridae*), respectively [100,108]. Horizontal infection by mosquito iridoviruses have also been investigated both for *Aedes taeniorhynchus* and *Cx. pipiens* mosquito larvae [109,110,111,112]. Mosquito Iridescent Virus (MIV), isolated from *Ae. taeniorhynchus* successfully infected healthy larvae when the cadaver of the infected larvae were added in the rearing water [109]. Diseased cadavers of larvae is thought to be the reservoir for MIV in nature, as MIV seemed to lose its infectivity after a couple of days in the water and in the soil [109] (Figure 1C). Another mosquito iridescent virus isolated from *Cx. pipiens* could infect the healthy larvae only at a very low rate, although this rate significantly increased following the addition of parasitic mermithid of mosquito larvae *Strelkovimermis spiculatus* (Nematoda) to the rearing water [111]. Together with the observed positive correlation between the presence of MIV in natural populations of *Cx. pipiens* [112], these results suggested the MIV is transmitted mainly by a mermithid vector to the mosquito larvae in nature [111] (Figure 1E). Mosquito densoviruses, Dipteran brevidensovirus 1 and Dipteran brevidensovirus 2, have also been experimentally shown to infect mosquito larvae when infected crushed larvae or cultured cells were added to the rearing water [113,114,115](Figure 1B). The stability and infectivity of Dipteran brevidensovirus 1 in water over a year suggested that the natural water bodies could be a reservoir for these densoviruses and the horizontal transmission could be the major transmission route for their maintenance in nature [113]. 

For mosquito specific RNA viruses, horizontal transmission during the larval stage is rarely studied experimentally and studies showed conflicting results [76,116]. Kamiti River Virus (KRV, *Flaviviridae*) was able to infect the larvae when added to rearing water, while CxFV (*Flaviviridae*) was not detected in the water where the infected larvae were reared [76,116]. A few studies on the diversity of RNA viruses also indirectly suggested the importance of horizontal transmission in nature, as many mosquitoes from the same collection site had genetically close virus variants independently from the host species [117,118]. 

Horizontal infection of adults through feeding has been tested only via infectious blood-feeding and only for some Flavivirus, Negevirus and Alphavirus strains. Infection through blood meal was not possible for tested Flaviviruses. For instance, CxFV did not infect *Cx. quinquefasciatus* mosquitoes fed with infectious blood [77] nor PCV was able to infect *Cx. annulirostris* [79]. For Eilat virus (Alphavirus) and Negev Virus (Negevirus) it was possible to successfully infect adults through blood meal but only when the virus titre was high [59,119]. Based on the close relationship of Negeviruses to plant viruses [44,59,120], suggestions have emerged that adult mosquitoes might also acquire the infection through feeding on plant nectar [121,122]. While feeding adult mosquitoes with infectious glucose or fructose has not yet been tested, the stability of a Negevirus has been shown in glucose over 70 days [123], highlighting the potential of the plant nectars to be the natural Negevirus reservoirs (Figure 1A). Alternatively, larvae might become infected through consumption of plant material [121] (Figure 1B). However, these hypotheses are pending to be verified. 

Overall, the mixed-mode transmission including both horizontal and vertical transmission routes is likely to be the key for MSV persistence and dispersal in nature, especially for the mosquito specific DNA viruses [124]. 

## 4. Interaction of MSVs with the Mosquito Immune System

### 4.1. MSVs and RNAi

The innate immune system of mosquitoes consists of a variety of different responses, including phagocytosis, nodulation, encapsulation as well as signalling pathways such as Janus kinase-signal transducer and activator of transcription (JAK-STAT) and the Toll and immune deficiency (Imd) pathways (reviewed in Reference [125]). However, RNA interference (RNAi) is thought to play the main role in antiviral defence.

RNAi is a nucleic-acid breakdown response that is divided into different pathways, which are characterised by the small RNAs produced during the process. Briefly, the microRNA (miRNA) pathway is involved in regulating endogenous gene expression, the Piwi-interacting RNA (piRNA) and endogenous small interfering RNA (siRNA) pathways mainly regulate transposon expression and the exogenous siRNA pathway is considered the main antiviral response in insects. Most knowledge about the RNAi pathways has originated from research in *Drosophila* and was extrapolated onto other insects, including mosquitoes (reviewed for example in Reference [126]). 

In the case of the siRNA pathway, this extrapolation from *Drosophila* data seems to be possible so far. In summary, long double stranded (ds)RNA derived from viral replication intermediates or secondary structures act as target for Dicer-2 (Dcr2). Dcr2 will cut the dsRNA into small interfering (siRNA), which are 21 nucleotides in size. These siRNA are in turn recognised by Argonaute-2 (Ago2), which becomes part of the so-called RNA-induced silencing complex (RISC), which uses one strand as a guide strand to find and degrade complementary (viral) RNA.

The piRNA pathway in *Drosophila* is mainly responsible for the control of transposon expression and focused mainly on the germline and surrounding cells. Briefly, long precursor RNA molecules are created from genomic transposon-rich clusters and cleaved by Zucchini (Zuc). This results in small RNAs (piRNAs), which can be identified by a sequence bias for uridine at 1st position of the sequence and are slightly longer than siRNAs (24–30 nt). This can be combined with further processing by PIWI family proteins, which results in the ping-pong-replication cycle, producing more piRNAs that are characterised by a bias for adenine at 10th position. These piRNAs are then shuttles to the nucleus or remain in the cytoplasm to continue the replication cycle [127].

In mosquitoes, there are major differences in terms of protein players, expression profiles and target sequences (reviewed in Reference [128]). Although an orthologue of Argonaute-3 is present in the mosquito genomes, none have been found for the PIWI family proteins found in *Drosophila*. Instead, there has been an extension of this family of proteins in *Culex*, *Aedes* and *Anopheles* [129,130,131]. Furthermore, piRNAs in mosquitoes have not only been concentrated on the germline but are also present in somatic tissue [101,127,132,133]. Lastly, piRNAs seem to also include viral targets [127,129,130,131,133,134,135,136].

The miRNA pathway is conserved among a variety of organisms, also including insects. Similarly to the siRNA pathway, the proteins involved in the miRNA pathway in mosquitoes are believed to be the same as found in *Drosophila* [137]. Very briefly, miRNA clusters in the nucleus are transcribed. This leads to the production of partial dsRNA stem loop structures, which are cleaved into 80 nts precursor (pre-)miRNAs. In the cytoplasm, these (pre-)miRNAs are again cleaved into 21/22 nts miRNA. These are bound to Argonaute-1 (Ago1) and shuttled to (partial) homologous sequences to induce degradation of translational inhibition [137].

Arbovirus-miRNA-pathway interactions have been reported, either by expression of viral miRNAs or changes in the mosquito-expressed miRNAs upon infection [126]. In contrast, data regarding the interaction of MSVs and the miRNA pathway in mosquitoes is limited to only one study suggesting no effects of MSV infection on miRNA expression [138] therefore this is not further discussed.

The production of siRNAs and piRNAs has been shown for a variety of MSVs. Most data concerns viruses that group into three viral families: *Flaviviridae*, *Birnaviridae* and *Phenuiviridae* (Table 3).

Studies on small RNAs induced by MSV infection in mosquitoes, so far highlighted the interaction between the mosquito RNAi pathways and MSVs, although whether the interaction is antiviral has not yet been thoroughly investigated. Nevertheless, there are clear indications in this direction with reports of viral clearance using repeated dsRNA transfection [142]. 

### 4.2. MSVs and RNAi Suppressor Molecules

Another clue for an antiviral interaction could also come from the detection of ways developed by the MSVs to surpass or block these pathways [146]. In fact, there are several RNAi suppressor molecules (VSRs) known, also for insect-specific viruses, that have been comprehensively reviewed elsewhere [147,148]. Very briefly, currently described VSRs have been shown to employ a selection of targets. However, the best characterised VSRs are RNA binding proteins, which are able to shield viral dsRNA from Dicer processing and subsequent RISC assembly. Examples here include the B2 proteins of nodaviruses (including Flock House virus, Wuhan nodavirus and Nodamura virus; *Nodaviridae*) as well as the 1A protein of the Drosophila C virus (*Dicistroviridae*), although the latter does not bind to short dsRNA such as siRNAs [149,150,151,152,153,154,155]. Interestingly, some B2 proteins of nodaviruses might also directly interact with Dicer to inhibit its function [151,156]. Targets of other VSRs can include direct interaction with Ago2 (thus blocking efficient target cleavage) or even degradation of dsRNA to prevent the formation of a mature RISC complex [153,157,158,159].

In comparison to other insects, relatively little data is available on VSRs against RNAi pathways in mosquitoes. Up until now, MSVs have only been investigated for the presence of VSR. Similar to the other viruses of the *Nodaviridae* family mentioned previously, the Mosinovirus encodes for a B2 protein that is capable of binding long dsRNA and thus inhibits processing of the dsRNA into siRNAs by Dicer [60]. The Culex Y virus (*Birnaviridae*) VP3 protein has been shown to exhibit similar properties. In addition, the VSR is able to also bind siRNAs, presumably preventing the efficient take up into the RISC complex [143,160]. 

## 5. Possible Uses of MSVs

Due to their special characteristics, like their inability to replicate in vertebrates or their pathogenicity against mosquitoes, some MSVs could be used in applications like vector control, vector-borne disease control, expression platforms and vaccines. MSVs are present in a wide variety and it can be expected that, depending on the application, different MSVs would be needed. This could include wildtype MSVs as well as genetically modified MSVs. Some possible application of MSVs are discussed in more detail below; however, more ideas/ applications could be developed in the future. 

### 5.1. Vector and Transmission Control 

Vector control and reduction/ inhibition of arbovirus infection are a broad-ranging topic and MSVs could be used to gain different outcomes. MSVs reducing the fitness, survival or fertility of mosquitoes could be used to control either a specific mosquito species (e.g. known vectors for certain arboviruses) or mosquitoes irrespective of their species. The specificity of the target species would depend on the host specificity of the MSVs used. Such an approach would reduce the overall mosquito burden and thereby arbovirus transmission; similar to the present vector control strategies (i.e. insecticide, fungus/ bacteria treatment). For instance, mosquito densovirus AaeDV successfully decreased general mosquito population density when added in artificial or natural ponds [161]. However, such an approach could face major problems like evolution of resistance or target specificity. In contrast, using MSVs that only affect the mosquito vector during the late-life stages or in the presence of an additional arbovirus infection could circumvent these problems as they would be more specific, would not interfere with the ecosystem and could be evolution-proof [162]. Some densoviruses, like AgDV, are examples for late life acting pesticides. Unlike other densoviruses, AgDV does not significantly replicate in mosquito larvae and pupae but replicates in older adults [163].

An approach where double infections of MSV and arboviruses are needed to affect the mosquito, would benefit from the fact that MSVs often result in persistent infections in mosquitoes, with little effects on the mosquito host. The additional arbovirus infection could destroy this balance due to for example increased usage of nutrients, space and induction of the immune response. 

Besides, targeting the mosquito vector itself, MSVs could also be used to interfere only with arbovirus infection and transmission. This strategy would benefit from the observations that some MSVs interfere/restrict arbovirus infection as discussed in detail above. Such a strategy would only focus on arbovirus transmission and not targeting the mosquito vector by itself, thereby being more ecosystem friendly. A critical point for such an approach would be the availability of MSVs interfering with a variety of arboviruses. Until now, no MSV has been identified that restricted arboviruses from all important arbovirus families. Most of the time, reported restriction was only observed when both MSV and arbovirus belonged to the same virus family. To estimate the presence of such an MSV, more knowledge is needed regarding the mechanism of arbovirus interference by the MSVs tested. Possible explanations could include superinfection exclusion, induction of the immune system, competition for nutrition and space; however, no final results have been reached for most tested MSVs so far. 

In addition to wildtype MSVs, genetically modified MSVs could be used for vector control. This includes MSVs expressing proteins, (i) inhibiting the immune system, (ii) stimulating the immune system, (iii) inhibiting the virus to infect the mosquito at all and (iv) targeting the virus directly. Reducing the immune system could be achieved by expressing dsRNA molecules targeting key proteins of the antiviral immune response, for example, Ago2 or Dcr2 of the RNAi response of the mosquito vector [164]. Reduced immune system would destroy the careful balance between arbovirus and mosquito and thereby lead to pathogenicity of the arbovirus instead of the typical persistent infection, believed to be important for a successful arbovirus transmission. On the flip side, increasing the immune system would enable the mosquito to successfully target and clear the arbovirus. To this end, genetically modified MSVs could be used as expression vectors for overexpression of key antiviral proteins, like Ago2 and Dicer2.

Production of ligands binding to receptors, which are essential for arbovirus entry into mosquito cells could stop arbovirus infection in mosquitoes. A more specific approach would be the expression of molecules directly affecting or targeting a certain arbovirus, for example dsRNA molecules.

In light of recent findings, MSVs could also have the potential to “immunize” mosquitoes, even with the possibility to pass this on to their offspring. This approach would use the recent findings that virus specific sequences (mainly from MSVs) have been found in mosquito genomes, which in turn produce virus specific small RNAs. At the moment, it is hypothesized that parts of MSVs (mainly with an RNA genome) are reverse transcribed into viral cDNA during virus infection, followed by incorporation in specific parts of the genome and thereby production of small RNAs of these sequences. In turn, these viral specific sequences are expected to enter the antiviral RNA interference pathway in mosquitoes and thereby enable the mosquito to target these viruses upon a new infection [126,165]. Due to the genomic incorporation, this could be passed on to the offspring. Incorporating parts of pathogenic viruses into MSVs, known to produce viral cDNA, incorporation, production of small RNAs of the integrated parts and inheriting this trade would result in a unique “vaccine” to “immunize” mosquitoes.

A disadvantage of such approaches would be the virus specificity. They would only be successful in case of the presence of a single arbovirus in a certain area. However, combining different viruses or incorporating parts of different viruses in MSVs or producing several dsRNA molecules would broaden the amount of arboviruses to be targeted. 

### 5.2. Gene Expression Platforms and Vaccines

In addition to vector and transmission control, MSVs can also be used as expression tools as some of them replicate to high titres and are considered safe due to their inability to infect and replicate in vertebrate cells. This and the similarity of some MSVs to pathogenic arboviruses has identified them as good vaccine candidates. Erasmus et al. used the insect specific alphavirus, Eilat virus backbone (non-structural proteins, essential for replication) to express proteins of arboviruses (structural proteins) from the genus alphavirus (e.g. Eastern and Venezuelan equine encephalitis viruses, Chikungunya virus). These chimeric viruses were used to immunize mice, resulting in protection against the pathogenic arboviruses [166]. This established the possible use of MSVs as vaccine backbones. Similar experiments could be imagined for the other two important groups of arboviruses—Flavivirus genus and bunyavirales order. However, a crucial point for such an application is the presence of a reverse genetic system for the MSV. Such systems have been reported for MSVs belonging to the alphavirus and the flavivirus genus [29,167,168]. In contrast, no functional reverse genetic system for MSVs belonging to the bunyaviruses has been published so far, although it was tried for at least some. 

### 5.3. Diagnostic Assays

Having the structural protein genes of the arboviruses and non-structural protein genes of MSVs, these chimeric MSVs are unable to replicate in vertebrate cells while being antigenically same as arboviruses. As such, chimeric (MSV-arbovirus) viruses could also be used in diagnostic assays, for example to determine the presence of antibodies against arbovirus proteins in blood samples. The advantage of such chimeric viruses compared to virus-like particles: (i) the even higher production, (ii) no vertebrate cells needed and (iii) better virus assembly. Chimeric viruses also have similar advantages to other production approaches of antigens, like recombinant protein production (problems with protein folding and loss of epitopes) or inactivated pathogenic viruses (loss of epitopes by inactivation). Thereby, chimeric viruses are expected to be cheaper, easier to produce and are expected to give a higher sensitivity of detection, due to correct folding and representation of epitopes, as recently shown for Chikungunya and Eilat virus chimera [169]. Moreover, commonly produced antigens of pathogenic arboviruses for diagnostic kits need to be produced in a high biocontainment laboratory, followed by concentration, purification and inactivation. In contrast, chimeric MSVs need no high biocontainment, inactivation and possibly neither concentration nor purification, thereby resulting in less costs and safety concerns. 

## 6. Conclusions and Future Directions

The research on MSVs has steadily grown in the last years, due to an increase in their discoveries and new potential uses, including arboviral disease control, diagnostic assays and vaccine development. Although, this has led to an increase in knowledge, a lot is still unknown and current data is largely contradictory. This is possibly due to different experimental set ups having significant effects on the outcomes (e.g. interference with arboviruses).

In the following years, more research needs to be performed to answer some of the urgent questions in MSV research. For instance, the interaction of MSVs with the mosquito, specifically the immune response, should be elucidated thoroughly to predict its use as an expression tool as well as its effect on the overall mosquito fitness. Another urgent question is the mechanisms of the interaction of MSVs with the arboviruses, which is still unknown. So far, the research into these interactions is often lacking in cells with an intact RNAi response as well as in vivo, which may result in different observations. Furthermore, most research to date is focused on Flaviviruses, while MSVs of other families are rarely studied. Moreover, more knowledge is needed regarding the mechanism of how MSVs are acquired and transmitted to set up a release and distribution plan to use MSVs as disease control tools. To date, the research showed that their transmission might differ between the different virus families and hosts, and could potentially vary even between virus strains. The efficiency of transmission can be affected by biotic and abiotic factors. Therefore, more studies are warranted to understand how these factors can change virus transmission, and how differences in virus transmission would affect the evolution and the interactions of the MSVs with the host and the host microbiota, including arboviruses. 

Finally, the future challenges will include not only understanding the interaction of MSVs with the mosquito host but also to determine the differences between the varieties of MSVs and to eventually identify and modify the useful MSVs in context of the different applications.

## Figures and Tables

**Table 1:** Mosquito-specific viruses discovered (confirmed to grow on mosquito but not mammalian cell lines).

MSV (Acronym)	Year	First Mosquito Host	Country Isolated	Family	Reference
				***Flaviviridae***	
Cell-fusing agent virus (CFAV)	1975	*Aedes aegypti* cell line	USA		[8]
Kamiti River virus (KRV)	2003	*Aedes macintoshi*	Kenya		[20]
Palm creek virus (PCV)	2013	*Coquillettidia xanthogaster*	Australia		[21]
Hanko virus (HANKV)	2014	*Ochlerotatus* sp.	Finland		[22]
Culex flavivirus (CxFV)	2007	*Culex pipiens*	Japan		[23]
Aedes flavivirus (AEFV)	2009	*Aedes albopictus, Aedes flavopictus*	Japan		[24]
Aedes galloisi flavivirus (AGFV)	2012	*Aedes galloisi*	Japan		[25]
Anopheles flavivirus (AnFV)	2018	*Anopheles gambiae*	Kenya		[26]
Mercadeo virus (MECDV)	2015	*Culex* sp.	Panama		[27]
Quang Binh virus (QBV)	2009	*Culex tritaeniorhyncus*	Vietnam		[28]
Nienokoue virus (NIEV)	2017	*Culex* sp.	Côte d’Ivoire		[29]
Culex theileri flavivirus (CTFV)	2017	*Anopheles vagus*	Indonesia		[30]
Nhumirim virus (NHUV)	2015	*Culex chidesteri*	Brazil		[31]
Spanish Culex flavivirus (SCxFV)	2012	*Culex pipiens*	Spain		[32]
Spanish Ochlerotatus flavivirus (SOcFV)	2012	*Ochlerotatus caspius*	Spain		[32]
Ochlerotatus caspius flavivirus (OCFV)	2013	*Ochlerotatus caspius*	Portugal		[33]
Mediterranean Ochlerotatus Flavivirus (MoFV)	2012	*Ochlerotatus caspius*	Spain		[32]
Ilomantsi virus (ILOV)	2014	*Ochlerotatus riparius* and/or *Anopheles* spp. (Pool; COI based identification)	Finland		[22]
Lammi virus (LAMV)	2009	*Aedes cinereus* (COI based identification)	Finland		[34]
Nounane virus (NOUV)	2009	*Uranotaenia mashonaensis*	Côte d’Ivoire		[35]
Chaoyang virus (CHAOV)	2013	*Aedes vexans nipponii*	South Korea		[36]
Barkedji virus (BJV)	2013	*Culex perexiguus*	Israel		[37]
T’Ho virus	2009	*Culex quinquefasciatus*	Mexico		[38]
Yamadai flavivirus	2015	*Culex mosquitoes*	Japan		[39]
Culiseta flavivirus	2016	*Culiseta melanura*	USA		[40]
Marisma mosquito virus (MMV)	2012	*Ochlerotatus caspius*	Spain		[32]
Nanay virus (NANV)	2013	*Culex (Melanoconion) ocossa*	Peru		[41]
Kampung Karu virus (KPKV)	2018	*Anopheles tessellatus*	Malaysia		[42]
Long Pine Key virus (LPKV)	2018	*Anopheles crucians*	USA		[42]
La Tina virus (LTNV)	2018	*Aedes scapularis*	Peru		[42]
				***Togaviridae***	
Eilat virus (EILV)	2012	*Anopheles coustani*	Israel		[11]
				***Bunyaviridae***	
Badu virus (BADUV)	2016	*Culex* sp.	Australia		[16]
Kibale virus (KIBV)	2013	*Culex simpliciforceps*	Uganda		[13]
Ferak virus (FERV)	2015	*Culex* sp.	Côte d’Ivoire		[15]
Phasi Charoen virus (PCLV)	2009	*Aedes aegypti*	Thailand		[43]
Cumuto virus (CUMV)	2014	*Culex portesi*	Trinidad and Tobago		[44]
Herbert virus (HEBV)	2013	*Culex nebulosus*	Côte d’Ivoire		[13]
Tai virus (TAIV)	2013	*Culicidae* sp.	Côte d’Ivoire		[13]
Jonchet virus (JONV)	2015	*Culex* sp.	Côte d’Ivoire		[15]
Gouleako virus (GOLV)	2011	*Culex* sp.	Côte d’Ivoire		[14]
Anadyr virus (ANADV)	2014	*Aedes* sp.	Russia		[45]
				***Rhabdoviridae***	
Aedes Anphevirus (AeAV)	2018	*Aedes albopictus* cell line RML-12	USA		[46]
Puerto Almendras virus (PTAMV)	2014	*Ochlerotattus fulvus*	Peru		[47]
Arboretum virus (ABTV)	2014	*Psorophora albigenu*	Peru		[47]
Culex tritaeniorhynchus rhabdovirus (CTRV)	2011	*Culex tritaeniorhynchus*	Japan		[48]
Merida virus (MERDV)	2016	*Culex quinquefasciatus*	Mexico		[49]
Moussa virus (MOUV)	2010	*Culex decens*	Côte d’Ivoire		[50]
Coot Bay virus almendravirus	2017	*Anopheles quadrimaculatus*	USA		[51]
Rio Chico virus almendravirus	2017	*Anopheles triannulatus*	Panama		[51]
Balsa almendravirus	2017	*Culex erraticus*	Colombia		[51]
				***Mesoniviridae***	
Houston virus (HOUV)	2018	*Culex quinquefasciatus*	Mexico		[52]
Nse virus (NseV)	2013	*Culex nebulosus*	Côte d’Ivoire		[53]
Meno virus (MenoV)	2013	*Uranotaenia chorleyi*	Côte d’Ivoire		[53]
Hana virus (HanaV)	2013	*Culex* sp.	Côte d’Ivoire		[53]
Dak Nong virus (DKNV)	2013	*Culex tritaeniorhynchus*	Vietnam		[54]
Yichang virus (YCV)	2017	*Culex* sp.	China		[19]
Casuarina virus (CASV)	2014	*Coquillettidia xanthogaster*	Australia		[55]
				***Tymoviridae***	
Culex originated *Tymoviridae*-like virus (CuTLV)	2012	*Culex* sp.	China		[56]
				***Birnaviridae***	
Culex Y virus (CYV)	2012	*Culex pipiens (s.l.)*	Germany		[57]
Espirito Santo virus (ESV)	2012	*Aedes albopictus* C6/36 cells	Brazil		[58]
				Negeviruses *	
Uxmal virus	2018	*Ochlerotatus taeniorhynchus*	Mexico		[52]
Mayapan virus	2018	*Psorophora ferox*	Mexico		[52]
Santana virus	2013	*Culex* sp.	Brazil		[59]
Wallerfield virus (WALV)	2014	*Culex portesi*	Trinidad & Tobago		[44]
Dezidougou virus	2013	*Aedes aegypti*	Côte d’Ivoire		[59]
Loreto virus	2013	*Anopheles albimanus*	Peru		[59]
Negev virus	2013	*Culex coronator*	USA		[59]
Piura virus	2013	*Culex* sp.	Peru		[59]
				***Nodaviridae***	
Mosinovirus (MoNV)	2014	*Culicidae* mosquitoes	Côte d’Ivoire		[60]
				***Reoviridae***	
Mangshi virus (MSV)	2015	*Culex tritaeniorhynchus*	China		[61]
Ninarumi virus (NRUV)	2017	*Ochlerotatus fulvus*	Peru		[30]
High Island virus (HISLV)	2017	*Psorophora ciliata*	USA		[30]
Banna virus (BAV)	2017	*Culicoides* sp.	China		[62]
Parry’s Lagoon virus (PLV)	2016	*Culex annulirostris*	Australia		[63]
Fako virus (FAKV)	2015	Mosquito pool	Cameroon		[64]
Aedes pseudoscutellaris reovirus (APRV)	2005	*Aedes pseudoscutellaris* mosquito cells	France		[65]
Cimodo virus (CMDV)	2014	*Culicidae* sp.	Côte d’Ivoire		[66]
				***Parvoviridae***	
Culex pipiens pallens densovirus (CppDV)	2008	*Culex pipiens pallens*	China		[67]
Culex pipiens densovirus (CpDV)	2000	*Culex pipiens pipiens*	France		[68]
Aedes albopictus densovirus 2 (AalDV 2)	1993	*Aedes albopictus* C6/36 cells	France		[69]
				***Iridoviridae***	
Anopheles minimus Iridovirus (AMIV)	2015	*Anopheles minimus*	China		[70]
				***Permutotetraviridae***	
Sarawak virus (SWKV)	2017	*Aedes albopictus*	Malaysia		[30]
Shinobi tetravirus (SHTV)	2018	*Aedes albopictus* C6/36 cells	Japan		[71]
				***Iflaviridae***	
Armigeres iflavirus	2017	*Armigeres mosquitoes*	Philippine		[72]
				***Orthomyxoviridae***	
Sinu virus	2017	Adult mosquito pool	Colombia		[73]
				***Totiviridae***	
Omono river virus	2011	*Culex* sp.	Japan		[74]

* proposed taxon [59].

**Table 2:** Experimental interaction of mosquito-specific viruses with arboviruses in literature.

MSV	Arbovirus	Year	Method		Effect on Growth of Arbovirus			Reference
			In-Vitro	In-Vivo	Reduction	Increase	No Effect	
CFAV	DENV-1 ZIKV	2019	Yes Yes	Yes Yes	Yes Yes	No No	No	[85]
NHUV	ZIKV DENV CHIKV	2018	Yes Yes Yes	Yes No No	Yes Yes No	No No No	No	[82]
MRV, SHTV	ZIKV	2018	Yes	No	Yes	No	No	[71]
CFAV, PCLV	ZIKV DENV LACV	2018	Yes Yes Yes	No No No	Yes Yes Yes	No No No	No	[83]
CxFV	RVFV	2018	No	Yes	No	No	Yes	[78]
AeAV	DENV	2018	Yes	No	Yes	No	No	[46]
CFAV	DENV	2017	Yes	No	No	Yes	No	[86]
PCV	WNV	2016	No	Yes	No	Yes	No	[79]
EILV	SINV, VEEV, EEEV, WEEV, CHIKV CHIKV	2015	Yes No	No Yes	Yes Yes	No No	No	[84]
CxFV	DENV, JEV JEV	2015	Yes Yes	No No	No No	No Yes	No	[87]
NHUV	WNV	2015	Yes	Yes	Yes	No	No	[81]
NHUV	WNV SLEV JEV	2014	Yes Yes Yes	No No No	Yes Yes Yes	No No No	No	[80]
PCV	WNV MVEV	2013	Yes Yes	No No	Yes Yes	No No	No	[21]
CxFV	WNV	2012	Yes	Yes	Yes	No	No	[76]
CxFV	WNV	2010	Yes	Yes	No	No	Yes	[77]
AalDV	JEV, DENV-2	2010	Yes	No	Yes	No	No	[88]
AalDV	DENV-2	2008	Yes	No	Yes	No	No	[89]
AalDV	DENV-2	2004	Yes	No	Yes	No	No	[90]

**AalDV:** Aedes albopictus densovirus, **AeAV:** Aedes Anphevirus, **DENV:** Dengue Virus, **CFAV:** Cell-fusing agent virus, **CxFV:** Culex Flavivirus, **EILV:** Eilat virus, **EEEV:** Eastern Equine Encephalitis Virus, **JEV:** Japanese Encephalitis Virus, **LACV:** La Cross Encephalitis Virus, **MRV:** Menghai rhabdovirus, **MVEV:** Murray Valley encephalitis virus, **NHUV:** Nhumirim virus, **PCLV:** Phasi charoen-like virus, **PCV:** Palm Creek virus, **RVFV:** Rift Valley Fever virus, **SHTV:** Shinobi tetravirus, **SINV:** Sinbis Virus, **SLEV:** St Louis Encephalitis virus, **VEEV:** Venezuelan Equine encephalitis virus, **WEEV:** Western equine encephalitis virus, **WNV:** West Nile Virus, **ZIKV**: Zika virus.

**Table 3:** Small RNA profile of MSVs in mosquitoes (in vitro and in vivo).

MSV	Family	Genome	Small RNAs	In Vivo /In Vitro	Species	Reference
Cell fusion agent virus (CFAV)	*Flaviviridae*	+ssRNA	piRNAs and siRNAs	in vitro	Aag2 (*Aedes aegypti*) C6/36 (*Aedes albopictus*)	[139,140]
			Small RNA **	in vitro	Aag2	[141]
Calbertado virus	*Flaviviridae*	+ssRNA	siRNAs	in vitro	CT (*Culex tarsalis*)	[142]
Palm Creek virus (PCV)	*Flaviviridae*	+ssRNA	siRNAs and piRNA-like	in vivo	*Aedes aegypti*	[138]
Culex Y virus (CYV)	*Birnaviridae*	dsRNA	piRNA-like	in vitro	Aag2 C7/10 (*Ae albopictus*) U4.4 (*Ae albopictus*)	[141]
			siRNAs	in vitro	CT Aag2 U4.4	[141,142,143]
Phasi-Charoen-like virus (PCLV)	*Phenuiviridae*	-ssRNA	Small RNAs **	in vitro	Aag2 Ae (*Ae aegypti*) C7/10	[141]
			siRNAs and piRNAs	in vitro	Aag2 CT	[142,144]
			siRNAs and piRNAs	in vivo	*Ae aegypti*	[145]
Aedes pseudoscutellaris reovirus	*Reoviridae*	dsRNA	Small RNAs **	in vitro	Ae	[141]
Flock House virus	*Nodaviridae*	+ssRNA	siRNA	in vitro	CT	[142]
Culex narnavirus 1	Narna-like ***	+ssRNA ***	siRNA	in vitro	CT	[142]
Aedes Anphevirus (AeAV)	n/a *	-ssRNA	piRNA and siRNA	in vitro	Aag2.*w*MelPop-CLA (*Wolbachia* strain *w*MelPop-CLA infected Aag2)	[46]
Aedes albopictus densovirus 2 (Dipteran brevidensovirus 2)	*Parvoviridae*	ssDNA	Small RNAs **	in vitro	Aag2	[141]
Aedes densovirus (Dipteran brevidensovirus 1)	*Parvoviridae*	ssDNA	siRNA	in vitro	Aag2	[142]

* Family as of yet unassigned. Order: Mononegavirales; ** Small RNAs have been isolated and mapped against the virus. The type of small RNA has not been identified; *** Based on classification of Reference [142].
